# ZFHX3 is indispensable for ERβ to inhibit cell proliferation via MYC downregulation in prostate cancer cells

**DOI:** 10.1038/s41389-019-0138-y

**Published:** 2019-04-12

**Authors:** Qingxia Hu, Baotong Zhang, Rui Chen, Changying Fu, Jun A, Xing Fu, Juan Li, Liya Fu, Zhiqian Zhang, Jin-Tang Dong

**Affiliations:** 1 Department of Genetics and Cell Biology, College of Life Sciences, Nankai University, 94 Weijin Road, 300071 Tianjin, China; 2 Southern University of Science and Technology, School of Medicine, 1088 Xueyuan Road, Shenzhen, Guangdong 518055 China; 3 Department of Hematology and Medical Oncology, Emory Winship Cancer Institute, Emory University School of Medicine, 1365C Clifton Road, Atlanta, GA 30322 USA

## Abstract

Both estrogen receptor 2 (ESR2, also known as estrogen receptor beta (ERβ)) and the zinc-finger homeobox 3 (ZFHX3, also known as ATBF1 for AT motif-binding factor 1) modulate prostate development and suppress prostatic tumorigenesis in mice. ZFHX3 is integral to proper functions of ESR1 (i.e., estrogen receptor alpha (ERα)), which belongs to the same family of proteins as ESR2, but is hardly expressed in prostate epithelial cells. It is not clear how ZFHX3 suppresses prostatic tumorigenesis. In this study, we investigated whether ZFHX3 and ERβ functionally interact with each other in the suppression of prostatic tumorigenesis. In two androgen receptor (AR)-positive prostate cancer cell lines, C4-2B and LNCaP, we first validated ERβ’s tumor suppressor activity indicated by the inhibition of cell proliferation and repression of MYC expression. We found that loss of ZFHX3 increased cell proliferation and MYC expression, and downregulation of MYC was necessary for ZFHX3 to inhibit cell proliferation in the same cell lines. Importantly, loss of ZFHX3 prevented ERβ from suppressing cell proliferation and repressing *MYC* transcription. Biochemically, ERβ and ZFHX3 physically interacted with each other and they both occupied the same region of the common *MYC* promoter, even though ZFHX3 also bound to another region of the *MYC* promoter. Higher levels of ZFHX3 and ERβ in human prostate cancer tissue samples correlated with better patient survival. These findings establish MYC repression as a mechanism for ZFHX3’s tumor suppressor activity and ZFHX3 as an indispensable factor for ERβ’s tumor suppressor activity in prostate cancer cells. Our data also suggest that intact ZFHX3 function is required for using ERβ-selective agonists to effectively treat prostate cancer.

## Introduction

Estrogen receptor 1 (ESR1) and 2 (ESR2), more commonly known as estrogen receptor alpha (ERα) and beta (ERβ), respectively, have diverse functions in a variety of tissues including the prostate^1^. While androgen and androgen receptor (AR) signaling is the driving force in prostatic carcinogenesis, estrogens and their receptors have also been implicated in the process^2^. ERβ, in particular, clearly plays important roles in both normal prostate development and prostatic tumorigenesis, including an inhibitory effect on the activity of AR signaling^2^. In normal prostates, whereas ERα is expressed in the stroma compartment, ERβ is predominantly expressed in the epithelium with a cellular localization to the nucleus^3–7^. ERβ is indeed essential for the differentiation of epithelial cells and the maintenance of the epithelium, as knockout of *Esr2* in mouse prostates causes neoplastic lesions such as hyperplasia and mouse prostatic intraepithelial neoplasia (mPIN)^6,8^. In addition, loss of ERβ is enough to convert epithelial cells to a mesenchymal state^9^, further indicating a role of ERβ in epithelial maintenance.

In prostatic tumorigenesis, ERβ primarily plays a suppressor role. In addition to the induction of mPIN by the loss of *Esr2* in mice^6,8^, ERβ suppresses cell proliferation, survival, and tumor growth in human prostate cancer cell lines^10,11^. While ERβ’s tumor suppressor activity appears to be ligand dependent^10,12–15^, it is androgen independent, because such an activity is detectable in both AR-positive and -negative prostate cancer cells^16^. In mouse prostate tumors induced by *Pten* deletion, downregulation of Esr2 has been detected^17^, which also supports a tumor suppressor function of Esr2 in prostate cancer. In human prostate cancer, ERβ signaling appears to inhibit cell survival of TMPRSS2–ERG tumors, which usually have a more aggressive clinical phenotype^18^; ERβ is downregulated in some tumors^4,7,19,20^; and a correlation has been observed between partial loss of ERβ and castration resistance^2^.

How ERβ exerts a tumor suppressor function in the prostate is not well understood, even though some mechanisms have been described. For example, ERβ can upregulate FOXO3A via PUMA to induce apoptosis^21^; interact with KLF5 and other transcription factors to enhance FOXO1 expression to induce anoikis in AR-negative prostate cancer cells^22^; and attenuate the transcriptional activity of AR in gene expression^23^. In addition, some cancer-causing molecules are transcriptionally repressed by ERβ, including the *MYC* oncogene^24,25^. Understanding how ERβ suppresses prostatic tumorigenesis is highly relevant to the development of therapeutic strategies in prostate cancer treatment^26^. For example, ERβ-selective agonists are promising agents in the treatment of prostate cancer, including the most lethal castration-resistant prostate cancer (CRPC), but outcomes have been inconsistent among different trials^27–31^. Mechanistic information should be helpful in improving the therapeutic outcomes.

The zinc-finger homeobox 3 (ZFHX3), also known as ATBF1 for AT motif-binding factor 1, is a large transcription factor containing 23 zinc-finger domains, 4 homeodomains, and multiple other motifs^32^. *ZFHX3* is frequently mutated in metastatic or high-grade human prostate cancers, and many of the mutations are frameshifting and thus function inactivating^33,34^. Specific deletion of *Zfhx3* in mouse prostates not only causes mPIN but also promotes mouse prostatic tumorigenesis induced by the loss of *Pten*^35,36^. Finally, in mouse prostate tumors induced by transgenic expression of Myc or an activating mutant of AR, downregulation of Zfhx3 has been observed^37,38^. Although these studies indicate a tumor-suppressive role of ZFHX3 in prostate cancer, it is unknown how ZFHX3 exerts such a suppressor function in prostate cancer.

Our previous studies have demonstrated that ZFHX3 modulates multiple hormonal signaling pathways, including those of estrogen (E_2_)-ER, progesterone-progesterone receptor (Pg-PR), and prolactin-prolactin receptor^39–41^. For example, deletion of *Zfhx3* in mouse prostates alters the expression of multiple molecules involved in E_2_ and Pg networks^35^; and ZFHX3 interacts with ERα to modulate its functions in gene regulation and cell proliferation control in breast cancer cells^39^. Considering that both Zfhx3 and ERβ are necessary for proper development of mouse prostates, both possess a tumor suppressor activity in prostate cancer, ZFHX3 interacts with ERα to modulate its functions, and ERβ is highly homologous to ERα; it is reasonable to propose that ZFHX3 and ERβ interact with each other to modulate cell proliferation and tumor growth of prostate cancer cells.

In this study, we tested whether and how ERβ and ZFHX3 coordinate to function in AR-positive prostate cancer cells. Like ERβ, ZFHX3 also suppressed cell proliferation and MYC expression in prostate cancer cells, and downregulation of MYC was necessary for the suppressive effect of ZFHX3 on cell proliferation. More importantly, ZFHX3 was in fact essential for ERβ to inhibit cell proliferation and MYC expression. Furthermore, ZFHX3 and ERβ interacted with each other to repress *MYC* transcription. These findings not only provide mechanistic insights into the tumor suppressor functions of ERβ and ZFHX3, they also have important implications for the application of ERβ-selective agonists in treating prostate cancer.

## Results

### Validation of ERβ’s tumor suppressor activity in the LNCaP and C4-2B prostate cancer cell lines

**Fig. 1:**
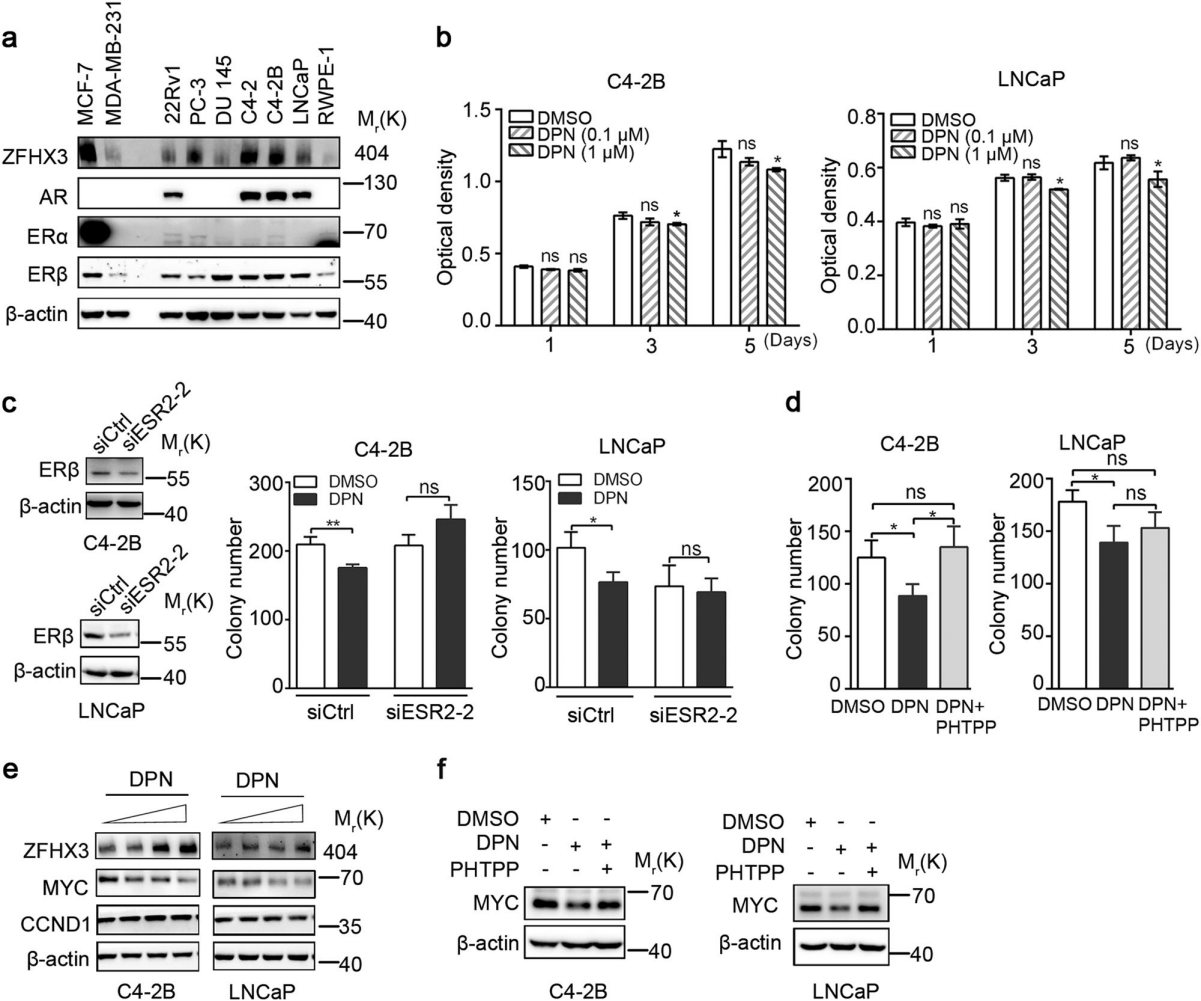
Activation of ERβ inhibits cell proliferation while upregulating ZFHX3 but downregulating *MYC* and *CCND1* in both C4-2B and LNCaP cell lines. **a** Expression of ZFHX3, AR, ERα, and ERβ in human prostate epithelial cell lines, as determined by Western blotting. Breast cancer cell lines MCF-7 and MDA-MB-231 were used as positive and negative controls, respectively, for ERα. **b** Treatment with DPN, an ERβ activator, decreased cell proliferation in two-dimensional (2D) culture in both C4-2B (left) and LNCaP (right) cell lines. Cells were serum-starved for 48 h before indicated DPN treatments. Optical densities represent cell numbers. *n* = 4. **c** Knockdown of ERβ eliminated the inhibitory effect of DPN on colony formation in both C4-2B (left) and LNCaP (right) cell lines in plates pre-coated with 0.35% soft agar. Cells were transfected with siESR2-2, which showed the highest efficiency of knockdown among the three siRNAs against *ESR2*, for 24 h and treated with DPN (0.1 µM) for 2 weeks. Colonies with a diameter > 100 µm were counted. Knockdown of ERβ was validated by Western blotting. *n* = 3. **d** Inhibition of ERβ function by its antagonist PHTPP eliminated the inhibitory effect of DPN on colony formation. *n* = 3. **e** DPN upregulates ZFHX3 expression but downregulates *MYC* and *CCND1* in C4-2B and/or LNCaP cells. DPN treatments were at 0, 0.01, 0.1, and 1 µM for 48 h, and protein expression was determined by Western blotting. **f** Inhibition of ERβ function by its antagonist PHTPP eliminated the inhibitory effect of DPN on MYC expression in C4-2B cells. DPN and PHTPP were at 0.5 μM. *P < 0.05; **P < 0.01; ns, not significant. ZFHX3, zinc-finger homeobox 3; AR, androgen receptor; ERα, estrogen receptor alpha; DPN, diarylpropionitrile; siRNA, small interfering RNA; PHTPP, 4-[2-phenyl-5,7-*bis*(trifluoromethyl)pyrazolo[1,5-*a*]pyrimidin-3-yl]phenol

We first surveyed the expression of ERα, ERβ, AR, and ZFHX3 in several prostate cancer cell lines by Western blotting (Fig. 1a). Compared to the ERα-positive MCF-7 breast cancer cells, ERα was hardly detectable in any of the prostate cancer cell lines tested. On the other hand, ERβ was expressed at moderate to high levels in most prostate cancer cell lines, including the androgen-sensitive LNCaP line and its androgen-insensitive derivative lines C4-2 and C4-2B. ZFHX3 expression was detectable in most of these cell lines as well, with C4-2, C4-2B, and LNCaP expressing higher levels, which is consistent with previous analysis of *ZFHX3* messenger RNA (mRNA)^34^. As expected, AR was expressed in LNCaP, C4-2, C4-2B, and 22Rv1 cell lines (Fig. 1a). Considering that LNCaP and C4-2B are AR positive and they both expressed higher levels of ERβ and ZFHX3, we chose these two cell lines for further analyses in this study.

We then tested the effects of ERβ on cell proliferation and target gene expression in C4-2B and LNCaP cells^13,42^. A known agonist of ERβ, diarylpropionitrile (DPN)^11,21,43,44^, was used to activate ERβ in hormone-deprived medium (phenol red free, 5% charcoal-stripped serum). In two-dimensional (2D) culture, the sulforhodamine B (SRB) assay showed that cell proliferation was reduced by DPN treatment at 1 µM for 5 days in both C4-2B and LNCaP cells (Fig. 1b). In the 3D soft agar colony formation assay, the number of colonies was decreased in both cell lines by DPN treatment at 0.1 μM (Fig. 1c), which was one-tenth of the effective concentration in the 2D SRB assay. To test the specificity of ERβ activation by DPN, we knocked down ERβ by RNA interference (RNAi), and found that knockdown of ERβ eliminated the inhibitory effect of DPN (Fig. 1c). We also used an antagonist of ERβ, 4-[2-phenyl-5,7-*bis*(trifluoromethyl)pyrazolo[1,5-*a*]pyrimidin-3-yl]phenol (PHTPP), to treat cells; as expected, PHTPP treatment eliminated the inhibitory effect of DPN on colony formation (Fig. 1d). These results indicate that the suppressive effect of ERβ on cell proliferation also occurs in AR-positive prostate cancer cells.

ERβ exerts its tumor suppressor role in prostate cancer cells by regulating gene transcription, including the inhibition of oncogenic *MYC* and *CCND1*^24,45,46^. As expected, activation of ERβ by DPN downregulated MYC in both C4-2B and LNCaP cells, and CCND1 in LNCaP cells (Fig. 1e), as detected by Western blotting. Moreover, inhibition of ERβ activity by the PHTPP antagonist diminished the inhibitory effect of DPN on MYC expression in both cell lines (Fig. 1f). Considering that ERα regulates the expression of ZFHX3^39,47,48^ and ERβ is homologous to ERα, we also examined whether ERβ has a similar effect. Interestingly, activation of ERβ by DPN significantly upregulated ZFHX3 expression in C4-2B cells, but not in LNCaP cells (Fig. 1e). Therefore, ERβ not only downregulates MYC in C4-2B and LNCaP cells but also upregulates ZFHX3 in C4-2B cells.

### Loss of ZFHX3 increases cell proliferation and colony/sphere formation in C4-2B cells

**Fig. 2:**
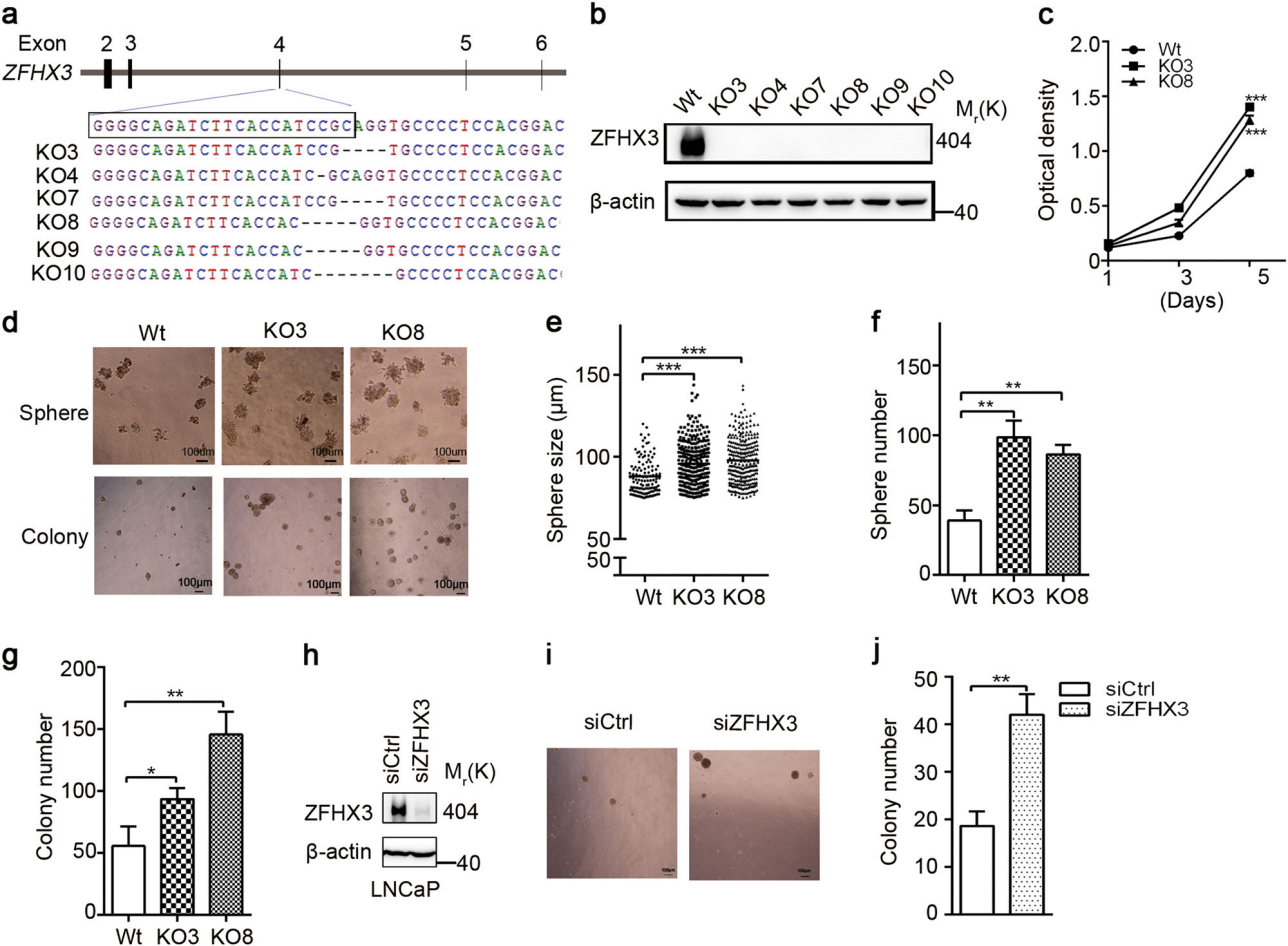
Loss of ZFHX3 increases cell proliferation, colony formation, and sphere formation in androgen-responsive prostate cancer cells. **a**, **b** Isolation of clones with successful CRISPR-Cas9-mediated truncating mutations of *ZFHX3* in C4-2B cells, as indicated by sequencing analysis of targeted locus (**a**) and expression detection of ZFHX3 by Western blotting (**b**). Wild-type sequence in parental cells is shown at the top of **a**. Wt, vector control cells; KO3, KO4, KO7, KO8, KO9, and KO10 are different clones of C4-2B cells with *ZFHX3* mutations. **c** Loss of ZFHX3 increased cell proliferation in two-dimensional (2D) culture in C4-2B cells. Optical densities represent cell numbers. *n* = 4. **d**–**g** Loss of ZFHX3 increased sphere formation in Matrigel (**d**—upper, **e**, **f**) and colony formation in soft agar (**d**—lower, **g**) in C4-2B cells. Cells were grown for 10–14 days in Matrigel or soft agar. Shown are bright field images of spheres (**d**, upper) and colonies (**d**, lower), range of sphere sizes (**e**), the average number of spheres with a diameter >75 µm per well (**f**), and the average number of colonies with a diameter >100 μm (**g**). The ImageJ program was used to determine sphere/colony sizes. **h**–**j** Knockdown of *ZFHX3* in LNCaP cells also increased sphere formation. The knockdown effect was validated by Western blotting (**h**), and bright field images of colonies (**i**) and the number of colonies with a diameter >100 μm (**j**) is shown. Scale bars in **d**, 100 μm. The *n* of **e**–**g**, **j** is 3. *P < 0.05; **P < 0.01; ***P < 0.001. ZFHX3, zinc-finger homeobox 3

Whereas the suppressive role of ZFHX3 in prostate cancer has been established in mouse knockout models^35,36^, the role of ZFHX3 has not been systematically examined in human prostate cancer cell lines. In this regard, we knocked out *ZFHX3* in the androgen-independent but not in androgen-responsive C4-2B cells using the CRISPR/Cas9 system. Clones with *ZFHX3* deletion were isolated and confirmed for *ZFHX3* disruption by sequence analysis (Fig. 2a) and Western blotting (Fig. 2b); and two clones (KO3 and KO8) were randomly selected from six confirmed clones and used for further analyses.

In the SRB assay, deletion of *ZFHX3* significantly increased cell proliferation (Fig. 2c), which is consistent with our previous finding in the AR-negative PC-3 cell line^34^. In the sphere formation assay in Matrigel, ZFHX3-null clones KO3 and KO8 not only formed larger spheres compared to the control clone (Fig. 2d—upper, e), they also formed significantly more spheres with a diameter >75 μm (Fig. 2f). In the soft agar assay, results were consistent, as KO3 and KO8 gave rise to many more colonies compared to the control clone (Fig. 2d—lower, g). In LNCaP cells, *ZFHX3* was knocked down by RNAi (Fig. 2h), and the knockdown also facilitated colony formation in soft agar (Fig. 2i, j). These results indicate that ZFHX3 plays a suppressive role in prostate cancer cells.

### Suppression of colony formation by ZFHX3 depends on the downregulation of MYC

**Fig. 3:**
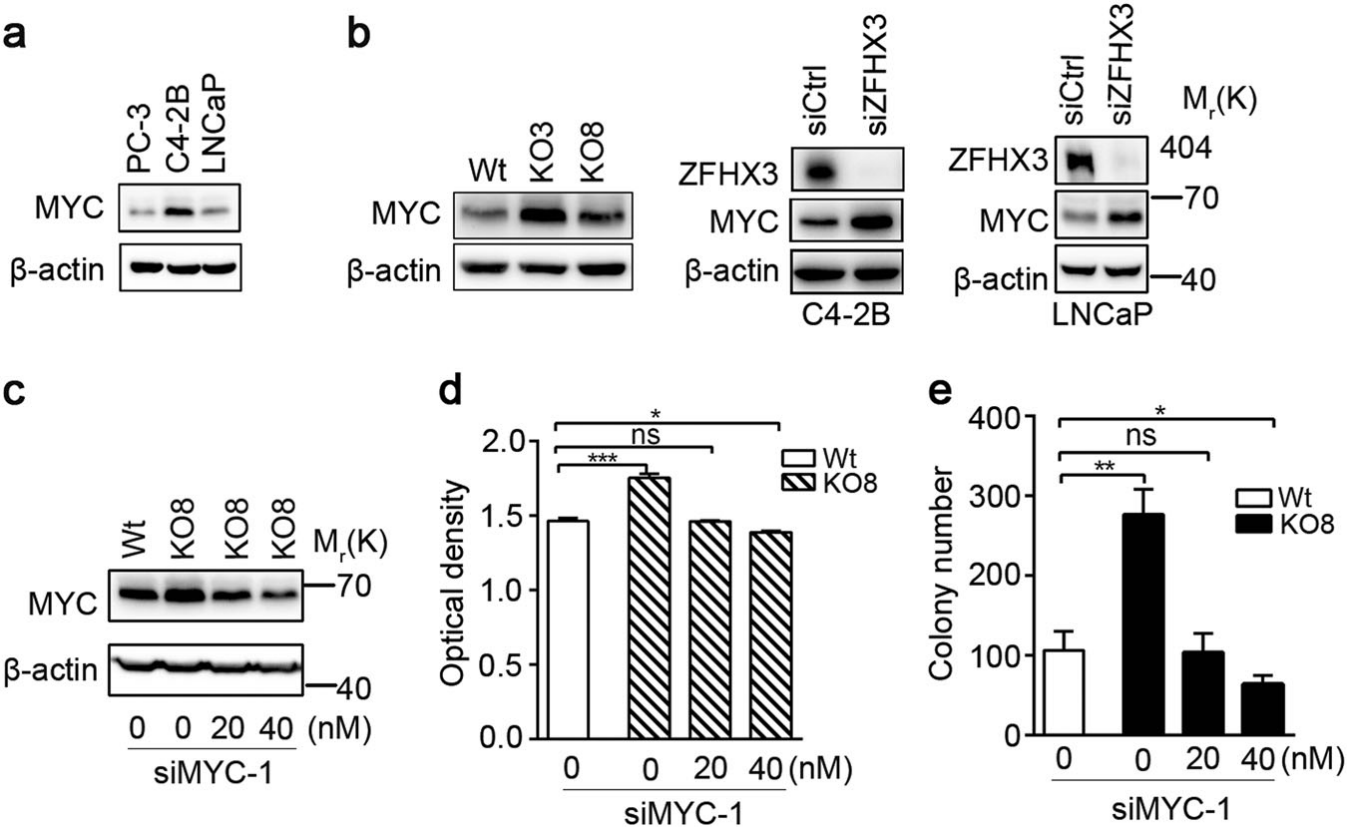
Inhibitory effect of ZFHX3 on colony formation depends on the downregulation of MYC in prostate cancer cells. **a** Expression of MYC in prostate cancer cell lines, as detected by Western blotting. **b** Knockout or knockdown of *ZFHX3* upregulated MYC expression in C4-2B and LNCaP cells, as detected by Western blotting. Wt, KO3, and KO8 are vector control and two ZFHX3-null clones of C4-2B. **c**–**e** Knockdown of *MYC*, by using siRNAs against *MYC* at two concentrations (20 and 40 nM), eliminated the promoting effect of ZFHX3’s loss on cell proliferation and colony formation in soft agar in C4-2B cells. The SRB assay was used to measure cell proliferation (**d**), while the soft agar assay was used for colony formation (**e**). The *n* of both **d** and **e** is 4. *P < 0.05; **P < 0.01; ***P < 0.001; ns, not significant. ZFHX3, zinc-finger homeobox 3; siRNA, small interfering RNA; Wt, wild type; SRB, sulforhodamine B

In human prostate cancer, *MYC* is frequently amplified and overexpressed, while *ZFHX3* is frequently deleted^49^. Some studies have suggested that *MYC* could be a target gene of ZFHX3^41,50^. In mouse prostates, overexpression of *Myc* downregulates *Zfhx3* expression while inducing neoplastic lesions^38^. Taken together with the fact that *MYC* is a transcriptional target of ERβ (Fig. 1)^24^ and our hypothesis that ERβ and ZFHX3 function together, we tested whether ZFHX3 also downregulates MYC to suppress cell proliferation. Among LNCaP, C4-2B, and PC-3 prostate cancer cell lines, MYC expression was apparently higher in C4-2B cells (Fig. 3a). Interestingly, deletion or knockdown of *ZFHX3* clearly increased MYC expression in C4-2B and LNCaP cells, as detected by Western blotting (Fig. 3b). We also detected three other ERβ transcriptional targets involved in cell proliferation, *FOXO3A*, *CCND1*, and *CDKN1B*^21,45,46^, in ZFHX3-null cells, and found that loss of ZFHX3 also downregulated FOXO3A (Fig. S1a). To evaluate whether MYC upregulation mediates the role of *ZFHX3* deletion in cell proliferation, we used two concentrations (20 and 40 nM) of *MYC* small interfering RNA (siRNA) to reduce MYC expression to a level similar to (20 nM) and lower than (40 nM) that of wild-type control (Wt) cells (Fig. 3c). The promoting effects of ZFHX3 loss on cell proliferation and colony formation were still abrogated by the subtle knockdown of *MYC* (Fig. 3d, e). These results indicate that MYC upregulation by the loss of ZFHX3 plays a causal role in the promotion of cell proliferation and colony formation in prostate cancer cells.

### ZFHX3 physically interacts with ERβ in prostate cancer cells

**Fig. 4:**
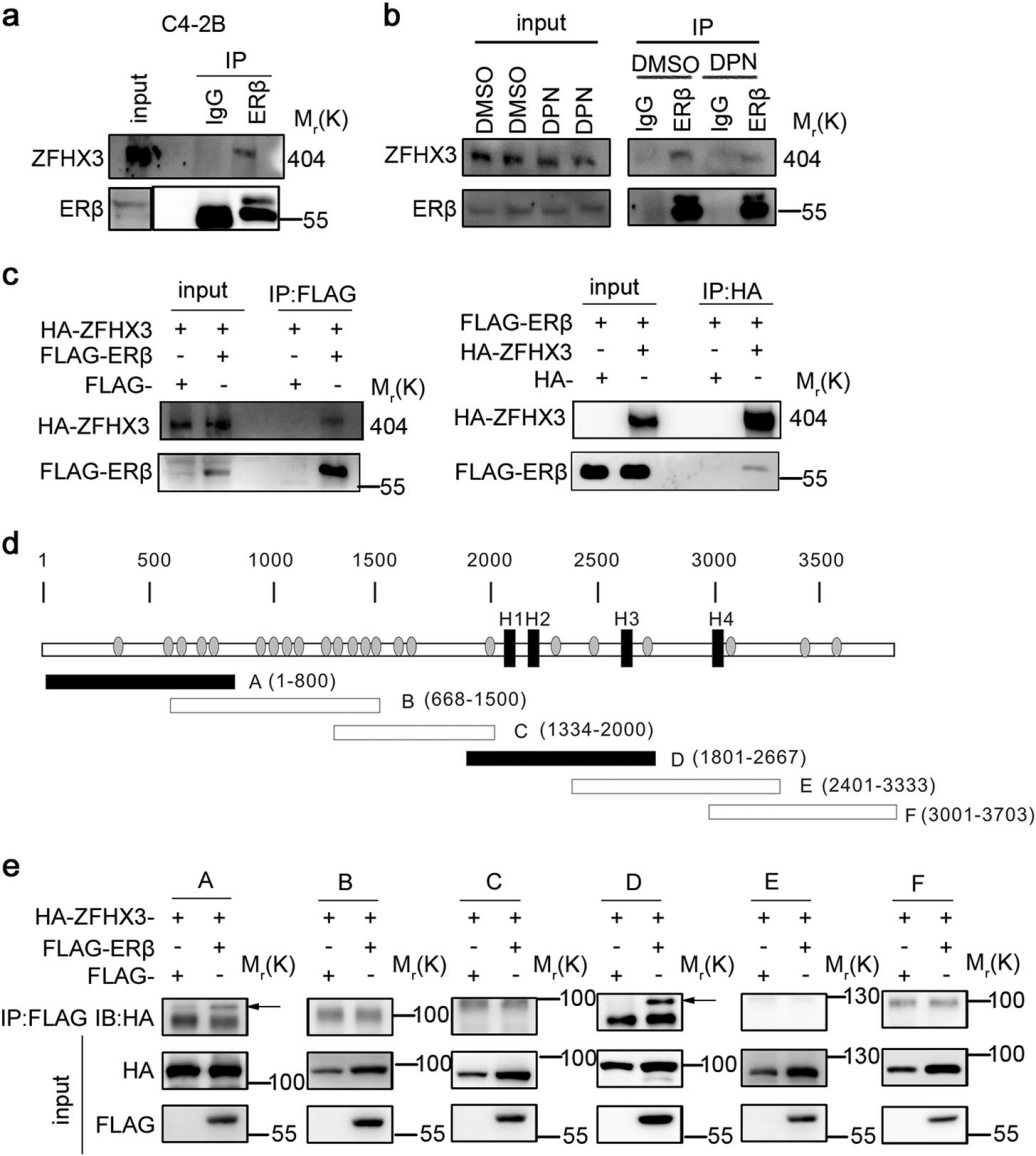
ZFHX3 and ERβ physically interact with each other independent of DPN treatment. **a** Cell lysates from C4-2B cells, which express both ZFHX3 and ERβ, were subjected to immunoprecipitation (IP) with anti-ERβ antibody and subsequent immunoblotting (IB) with anti-ZFHX3 antibody. Input indicates cell lysate not subjected to IP. **b** C4-2B cells were grown in phenol red-free medium with 5% charcoal-stripped FBS for 48 h and then treated with DPN for 48 h. IP and IB were performed as in **a**. **c** Expression plasmids for HA-tagged ZFHX3 (HA-ZFHX3) and FLAG-tagged ERβ (FLAG-ERβ) were transfected into 293T cells. Lysates were subjected to IP with anti-FLAG or anti-HA affinity gel, and then to IB with anti-HA or anti-FLAG antibody. **d** Schematic of full ZFHX3 protein (3703 residues, horizontal bar) with 23 zinc fingers (gray ovals) and 4 homeodomains (black rectangles). The six shorter bars below indicate six overlapping fragments of ZFHX3, named A to F. Each of the six fragments was tagged with HA, expressed in 293T cells, and tested for their interactions with ERβ by IP and IB. The two confirmed interactions with ERβ, A and D, are shown in solid dark. **e** IP and IB results for the interaction of FLAG-tagged ERβ and each of the six HA-tagged ZFHX3 fragments. The same procedures as in **c** were used. Arrows indicate the two fragments that were pulled down by ERβ (i.e., **a** and **d**). ZFHX3, zinc-finger homeobox 3; ERβ, estrogen receptor beta; FBS, fetal bovine serum; DPN, diarylpropionitrile

ZFHX3 interacts with ERα, and the interaction involves via the NR-box motif (LXXLL) of ZFHX3^39^. Considering that ERβ and ERα belong to the same protein family, sharing 97% similarity in their DNA-binding domains and 59% in their ligand-binding domains, it is possible that ZFHX3 also binds to ERβ in prostate cancer cells. To test this possibility, we performed immunoprecipitation (IP) and immunoblotting (IB) assays in C4-2B cells. In the ERβ protein complexes pulled down by ERβ antibody, ZFHX3 was detected (Fig. 4a). Similar results were obtained when C4-2B cells were treated with DPN (Fig. 4b). These results suggest that endogenous ZFHX3 and ERβ physically interact with each other regardless of ERβ status. We also expressed HA-tagged ZFHX3 (HA-ZFHX3) and FLAG-tagged ERβ (Flag-ERβ) in 293T cells and performed IP and IB with FLAG and HA antibodies. In the FLAG-ERβ complexes, HA-ZFHX3 was detected, and vice versa (Fig. 4c), further indicating an interaction between ZFHX3 and ERβ.

We also expressed six overlapping fragments of ZFHX3 (Fig. 4d), which were all HA-tagged and prepared and used for mapping ZFHX3 domains interacting with ERα^39^, along with FLAG-tagged ERβ in 293T cells. IP and IB demonstrated that two of the six fragments, A and D, interacted with ERβ (Fig. 4e). We noticed that fragment D of ZFHX3 contained the consensus NR box and ten of its variants and fragment A contained four of its variants. The NR box and one or more of its various could be specifically recognized by ERβ and thus mediate the ZFHX3-ERβ interaction. These results further support the ZFHX3-ERβ interaction, and also define the regions of ZFHX3 involved in the interaction.

### ZFHX3 and ERβ cooperate to repress the transcription of *MYC*

**Fig. 5:**
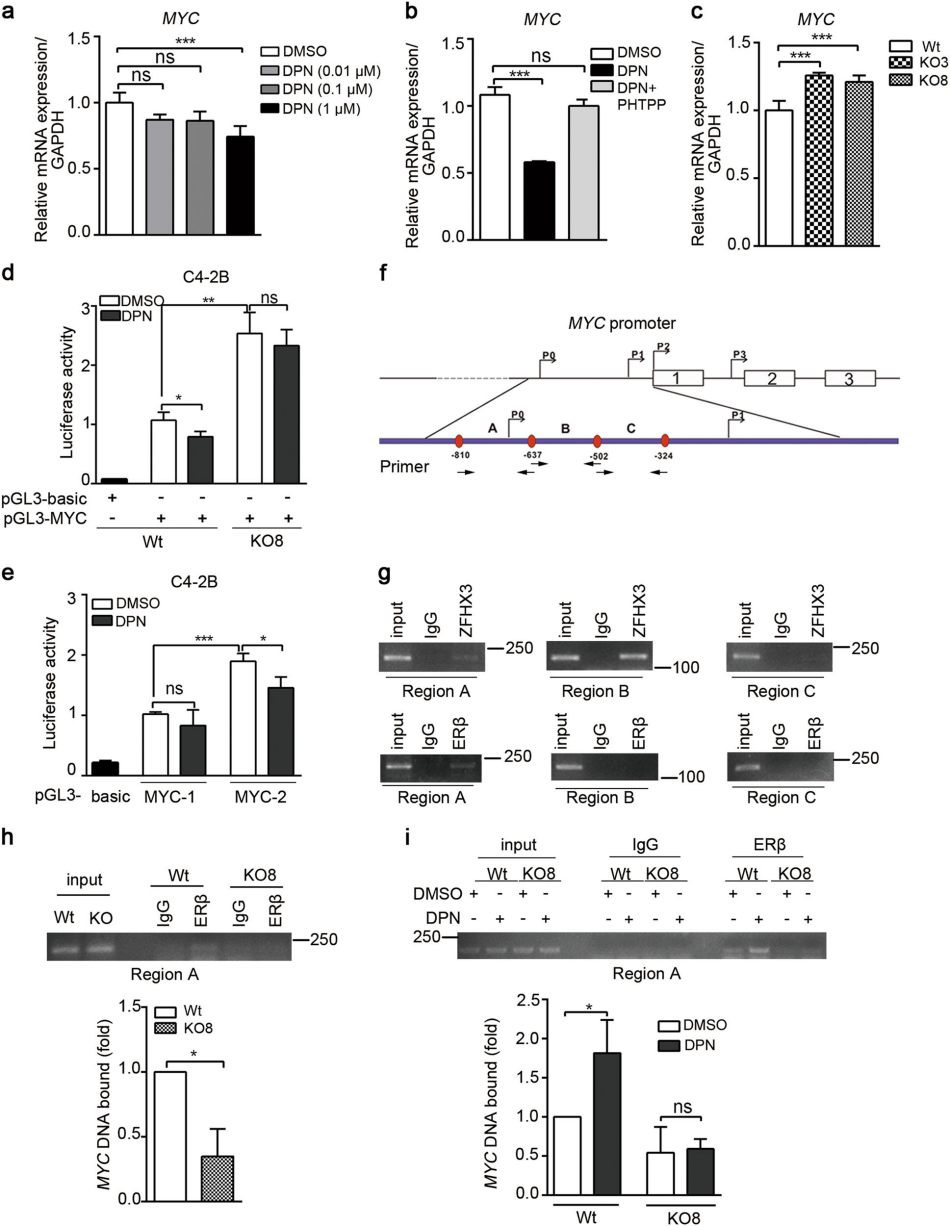
ZFHX3 and ERβ bind to *MYC* promoter to coordinately repress its transcription. **a** DPN treatment decreased *MYC* mRNA level in C4-2B cells. Hormone-deprived medium was used for DPN treatment. *n* = 4. **b** Inhibition of ERβ function by its antagonist PHTPP eliminated the inhibitory effect of DPN on *MYC* mRNA expression in C4-2B cells. DPN and PHTPP were at 0.5 µM. *n* = 4. **c** Knockout of *ZFHX3* increased *MYC* mRNA level. Wt, control clone; KO3 and KO8, two ZFHX3-null clones of C4-2B cells. *n* = 4. **d**, **e** DPN decreased the activity of *MYC* promoter. Expression plasmids of pGL3 vector control (pGL3-basic), pGL3 with *MYC* full-length promoters (**a**, pGL3-MYC with bases −2455 to 309) or pGL3 with two smaller MYC promoter fragments (**d**, pGL3-MYC-1 with bases −2024 to −1193 and pGL3-MYC-2 with bases −1200 to −200), and the pRL-TR reporter were transfected into C4-2B cells in phenol red-free medium supplemented with 2% CS-FBS. Twenty-four hours later, DPN treatments (0.1 µM, 48 h) were applied, and relative luciferase activities were then determined. *n* = 4. **f** Schematic of the *MYC* promoter region from base −1200 to base −200 relative to the P2 transcriptional initiation site (TIS), with locations of all four TISs, the first 3 exons, and primers used to amplify promoter regions A–C. Arrows under the promoter indicate primer locations. **g** Detection of ZFHX3- and ERβ-bound *MYC* promoter DNA in parental C4-2B cells using ChIP and regular PCR. **h**, **i** Binding of ERβ to *MYC* promoter region A in the presence (Wt) and absence (KO8) of ZFHX3 (**h**), with or without DPN treatment (**i**), using ChIP and regular PCR (upper) or real-time PCR (lower) in Wt and KO8 clones of C4-2B cells. The *n* of **h**, **i** is 3. *P < 0.05; **P < 0.01; ***P < 0.001; ns, not significant. ZFHX3, zinc-finger homeobox 3; ERβ, estrogen receptor beta; mRNA, messenger RNA; PHTPP, 4-[2-phenyl-5,7-*bis*(trifluoromethyl)pyrazolo[1,5-*a*]pyrimidin-3-yl]phenol; Wt, wild type; CS-FBS, calf serum-fetal bovine serum; DPN, diarylpropionitrile, ChIP, chromatin immunoprecipitation

Both ERβ and ZFHX3 are transcription factors that regulate MYC expression (Figs. 1 and 3), ERβ represses *MYC* transcription, and they interact with each other (Fig. 4)^34^. It is thus likely that ZFHX3 and ERβ coordinate to repress *MYC* transcription. To test this prediction, we first examined the effect of DPN treatment on *MYC* mRNA expression in C4-2B cells using real-time PCR. A higher concentration of DPN (1 μM) significantly reduced *MYC* mRNA level in C4-2B cells (Fig. 5a). Moreover, inhibition of ERβ activity by the PHTPP antagonist reversed the inhibitory effect of DPN on *MYC* mRNA expression in C4-2B cells (Fig. 5b). Loss of ZFHX3, on the other hand, increased *MYC* mRNA level (Fig. 5c), which is consistent with MYC protein expression under the same conditions (Fig. 3).

We next conducted a luciferase promoter activity assay to test whether ZFHX3 and ERβ interact to repress *MYC* transcription. A longer promoter of *MYC* (2764 bp, from −2455 to +309 bp) and two of its shorter fragments were cloned into the pGL3 luciferase reporter plasmid. The longer promoter had luciferase activity in control C4-2B cells, but the deletion of *ZFHX3* not only dramatically increased the activity but also eliminated the inhibitory effect of DPN on the activity (Fig. 5d), consistent with the real-time PCR results (Fig. 5a, c). In parental C4-2B cells, we also transfected two shorter *MYC* promoter reporter plasmids, pGL3-MYC-1 and pGL3-MYC-2 (bases −2024 to −1193 for pGL3-MYC-1 and −1000 to +200 for pGL3-MYC-2). The activity of pGL3-MYC-2 was higher than that of pGL3-MYC-1, and DPN significantly inhibited the activity of pGL-MYC-2 (Fig. 5e). These results indicate that ZFHX3 is involved in the function of DPN/ERβ signaling in the transcription of *MYC*.

To further define the role of ZFHX3 in *MYC* transcription, we explored the key regions of *MYC* promoter that are bound by ZFHX3 and ERβ using chromatin immunoprecipitation-PCR (ChIP-PCR). Based on the finding that higher promoter activity was detected with pGL3-MYC-2, we designed three pairs of PCR primers within the MYC-2 promoter region for PCR amplification (Fig. 5f). ChIP-PCR analyses showed that ZFHX3 bound to both A and B regions of the *MYC* promoter, while ERβ bound only to the A region (Fig. 5g). When *ZFHX3* was deleted, the binding of ERβ to the *MYC* promoter was dramatically reduced in C4-2B cells under normal culture conditions (without DPN, Fig. 5h); and DPN-increased ERβ binding to *MYC* promoter was eliminated under hormone-deprived conditions (Fig. 5i). These results indicate that both ZFHX3 and ERβ bind to the *MYC* promoter, and the binding of ERβ depends on the presence of ZFHX3.

### ZFHX3 is indispensable for ERβ to suppress cell proliferation and MYC expression

**Fig. 6:**
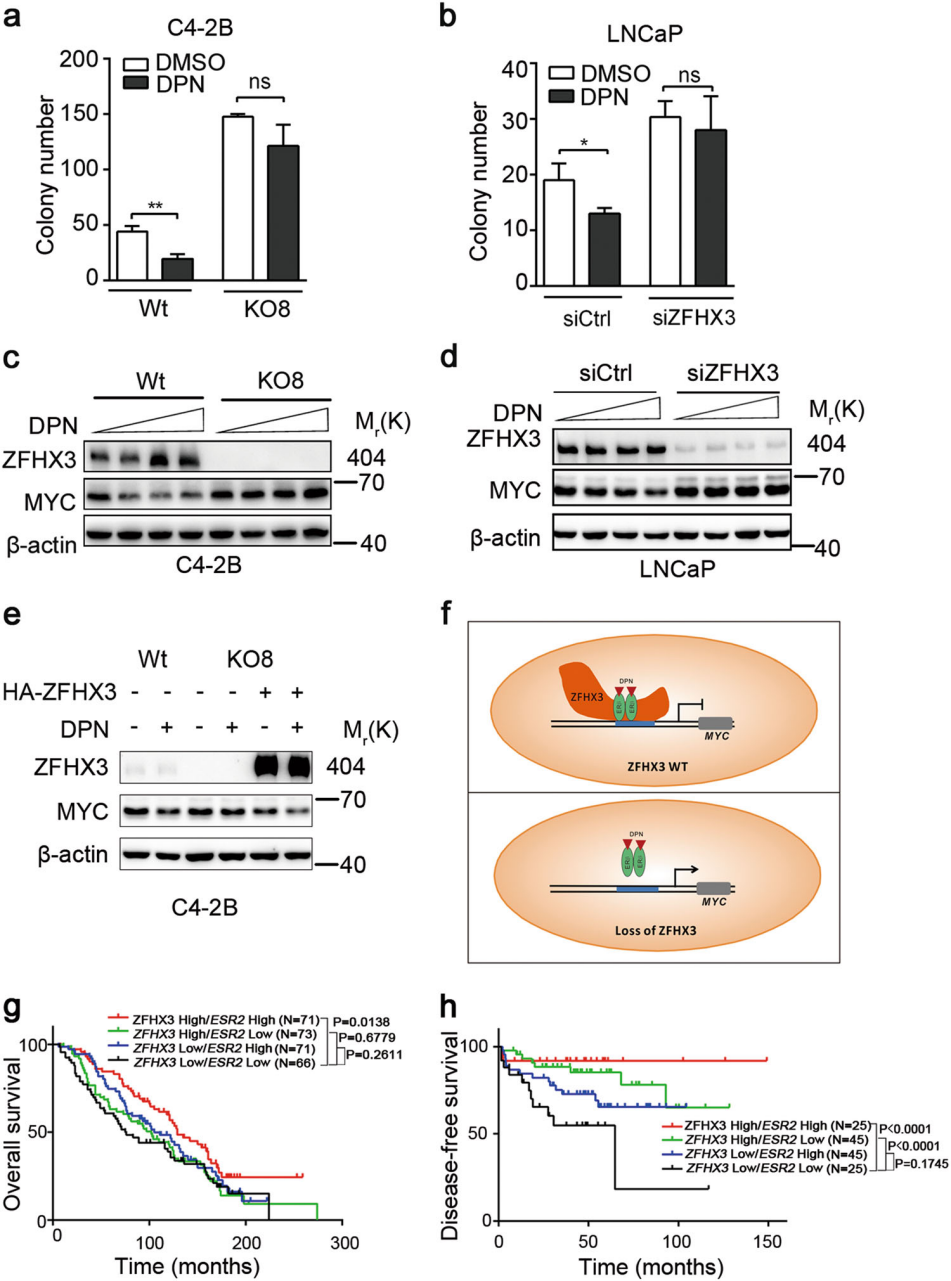
Loss of ZFHX3 eliminated the inhibitory effect of ERβ on colony formation and MYC expression in prostate cancer cells and correlated with worse patient survival. C4-2B (**a**, **c**) and LNCaP (**b**, **d**) cells were used for both colony formation assay (**a**, **b**) and MYC expression analysis (**c**, **d**). In colony formation assay, cells plated on 0.35% soft agar in phenol red-free medium were cultured, and colonies >100 µm were counted. MYC protein was detected by Western blotting. DPN was added to enhance the ERβ activity. **e** Transfection-mediated re-expression of ZFHX3 in the ZFHX3-null KO8 clone of C4-2B cells decreased MYC expression, as detected by Western blotting. **f** A model for how ZFHX3 is indispensable for ERβ to suppress cell proliferation and tumor growth in prostate cancer cells. In the presence of ZFHX3, ERβ interacts with ZFHX3 to repress the transcription of *MYC* and other oncogenes, but this repression is eliminated by the loss of ZFHX3. **g**, **h** Kaplan–Meier analysis of overall survival (**g**) and disease-free survival (**h**) of prostate cancer patients with different statuses of *ZFHX3* and *ESR2* expression. The *n* of **a**, **b** is 3. *P < 0.05; **P < 0.01; ns, not significant. ZFHX3, zinc-finger homeobox 3; ERβ, estrogen receptor beta; DPN, diarylpropionitrile

Considering that ZFHX3 is necessary for ERβ to repress *MYC* transcription (Fig. 5), it is reasonable to propose that ERβ also depends on ZFHX3 to exert its tumor suppressor activity. We tested this hypothesis using colony formation and MYC expression as indicators. Compared to control cells with normal ZFHX3 expression, in which DPN treatment (0.1 µM) significantly inhibited colony formation, loss of ZFHX3 in C4-2B cells (Fig. 6a), and knockdown of *ZFHX3* in LNCaP cells (Fig. 6b) not only increased colony formation but also attenuated the inhibitory effect of DPN on colony formation. Similar effects were detected on MYC expression, as loss of ZFHX3 in C4-2B cells (Fig. 6c) and knockdown of *ZFHX3* in LNCaP cells (Fig. 6d) prevented DPN from inhibiting MYC expression. Consistently, in ZFHX3-null KO8 cells, transfection-induced ZFHX3 expression not only reduced MYC expression but also sensitized cells to the inhibitory effect of DPN on MYC expression (Fig. 6e). These results indicate that ZFHX3 is indispensable for the tumor suppressor activity of ERβ in prostate cancer cells (Fig. 6f).

### Higher levels of ZFHX3 and ERβ correlate with better patient survival in prostate cancer

In two cohorts of prostate cancer patients in which data are available for both gene expression and overall survival^51^ or disease-free survival (DFS)^52^, we examined the correlation between *ZFHX3* and *ESR2* expression statuses with overall or disease-free survival. Patients were stratified into four groups according to the median levels of *ZFHX3* and *ESR2* expression: *ZFHX3* high/*ESR2* high, *ZFHX3* high/*ESR2* low, *ZFHX3* low/*ESR2* high, and *ZFHX3* low/*ESR2* low. Kaplan–Meier survival analysis demonstrated that when *ZFHX3* expression was higher, patients with higher *ESR2* had significantly better overall survival (Fig. 6g). Patients with higher *ZFHX3*, regardless of *ESR2* expression status, significantly correlated with better DFS (Fig. 6h). When *ZFHX3* was lower, however, even higher *ESR2* did not show a significant correlation with either overall survival or DFS (Fig. 6g, h), which is consistent with the notion that ZFHX3 is indispensable for the tumor suppressor function of ESR2.

## Discussion

In this study, we examined the role of ERβ and ZFHX3 in and their interaction as a mechanism for the regulation of proliferation of AR-positive prostate cancer cells. Distinct from the oncogenic function of ERα in breast cancer, ERβ is abundantly expressed in the prostate and plays a tumor suppressor role in prostate cancer^27,46,53,54^. On the other hand, MYC appears to be involved in the functions of both ERα and ERβ, although *MYC* is upregulated by ERα in breast cancer cells and downregulated by ERβ in prostate cancer cells^21,24,55^. Using two AR-positive prostate cancer cell lines, C4-2B and LNCaP, we demonstrated that ERβ also has a suppressive activity in these cell lines, as indicated by SRB and colony formation assays (Fig. 1). Such a suppressive activity has been previously detected in AR-negative PC-3 and DU 145 prostate cancer cell lines^22^. Interestingly, analysis of two ERβ target genes involved in cell proliferation control, *MYC* and *CCND1*, showed that MYC is clearly downregulated by ERβ in the inhibition of cell proliferation in C4-2B and LNCaP cells (Fig. 1). While validating the suppressive activity of ERβ in AR-positive prostate cancer cells, these findings also indicate that downregulation of MYC also mediates ERβ’s tumor suppressor activity in AR-positive prostate cancer cells.

Our findings in this study have established ZFHX3 as an indispensable factor for the tumor suppressor activity of ERβ. The most supportive evidence is that loss or downregulation of ZFHX3 in C4-2B or LNCaP cells eliminated the inhibitory effect of DPN/ERβ on colony formation and MYC expression (Fig. 6). Consistent with this, re-expression of ZFHX3 in ZFHX3-null cells sensitized cells to the inhibitory effect of DPN/ERβ on MYC expression (Fig. 6). Further supporting the necessity of ZFHX3 for ERβ’s tumor suppressor activity, we found that ERβ and ZFHX3 cooperate to repress the transcription of *MYC*. For example, ERβ physically interacts with ZFHX3 in prostate cancer cells via multiple domains of ZFHX3, as revealed by IP and IB analyses (Fig. 4); the same promoter site of *MYC* can be bound by both ERβ and ZFHX3, and loss of ZFHX3 prevented ERβ from binding to the site (Fig. 5). Loss of ZFHX3 also reduced the expression of FOXO3A (Fig. S1a), an apoptosis-promoting factor that is upregulated by ERβ^21^. Therefore, ZFHX3 inhibits cell proliferation likely by regulating multiple genes including both *MYC* and *FOXO3A*.

At present, it is unknown which other transcription factors are involved in the ERβ-ZFHX3 interaction. For example, a previous study demonstrated that ERβ interacts with KLF5 and CBP to induce FOXO1 transcription to suppress the proliferation of AR-negative prostate cancer cells^21^, but it is unknown whether KLF5 and CBP are also involved in the ERβ-ZFHX3 interaction in AR-positive prostate cancer cells. This mechanism is in addition to the previously reported mechanisms for ERβ, including the interaction with KLF5 and other co-factors to enhance FOXO1 expression to induce anoikis^22^.

ZFHX3 has been established as a tumor suppressor in prostate cancer in our previous studies, as its gene undergoes frequent somatic mutations in advanced prostate cancer^33,34^ and its deletion in mouse prostates causes neoplastic lesions and promotes *Pten* deletion-induced tumorigenesis^35,36^. Findings in this study provide additional evidence for a tumor suppressor activity of ZFHX3 in AR-positive prostate cancer cells, as indicated by assays of SRB, colony formation in soft agar, and sphere formation in Matrigel (Fig. 2).

Although ZFHX3 is clearly tumor suppressive in prostate cancer, how it exerts its tumor suppressor function was unknown. Our findings indicate that being part of ERβ signaling is an important mechanism for ZFHX3’s tumor suppressor activity in prostate cancer, as ZFHX3 is clearly upregulated by activated ERβ in C4-2B cells (Fig. 1), and repression of *MYC* transcription by ERβ required the interaction of ERβ with ZFHX3 (Figs. 4 and 5). However, ERβ is not required for ZFHX3 to suppress cell proliferation, as loss of ZFHX3 alone increased the formation of colonies and spheres in complete medium (Fig. 2); and even in the absence of hormones (charcoal-stripped serum and phenol red-free medium), loss of ZFHX3 still increased colony formation (Fig. 6). Therefore, other unknown mechanisms are also responsible for ZFHX3’s tumor suppressor activity in prostate cancer cells.

MYC is a well-established oncoprotein that plays a driving role in the development and progression of multiple types of cancers including prostate cancer. For example, *MYC* is frequently amplified and overexpressed in human prostate cancer^49^, and transgenic overexpression of *Myc* in mouse prostates not only induces prostate tumors but also promotes tumor progression^38^. In human malignancies, genomic amplification is one mechanism for *MYC*’s overexpression, but abnormalities in multiple signaling pathways also upregulate *MYC* expression^56^. Although ERβ clearly downregulates *MYC* in prostate cancer cell lines^24^ and *MYC* promoter contains consensus ZFHX3 binding sites^50^, our findings for the first time demonstrate that *MYC* transcription is indeed repressed by ZFHX3 in prostate cancer cells. For example, loss of ZFHX3 dramatically increased MYC expression in both C4-2B and LNCaP prostate cancer cells (Fig. 3), and ZFHX3 clearly bound to the *MYC* promoter (Fig. 5). Although one region of the *MYC* promoter was bound by both ZFHX3 and ERβ, another region was only bound by ZFHX3 and not by ERβ (Fig. 5), further indicating that ZFHX3 is a bona fide repressor of *MYC* transcription in prostate cancer cells.

Importantly, repression of *MYC* indeed plays a causal role in ZFHX3-mediated suppression of cell proliferation in prostate cancer, as silencing MYC in C4-2B cells prevented *ZFHX3* deletion from increasing cell proliferation in both SRB and colony formation assays (Fig. 3), and this remained true even when *MYC* expression was slightly reduced to a level comparable to that of control cells (Fig. 3). Therefore, MYC upregulation plays a causal role in the promotion of cell proliferation and colony formation by the loss of ZFHX3 in prostate cancer cells.

ERβ-selective agonists have been tested as therapeutic agents in the treatment of prostate cancer, including the most lethal form, CRPC^27–31^, and because ERβ has tumor suppressor activity, it is frequently downregulated^4,7,19,20,27,53,54^, partial loss of ERβ correlates with castration resistance^2^, and such agonists upregulate ERβ expression^57^. Indeed, an inhibitory effect of ERβ agonists on AR activity, cell proliferation, and tumor growth have been demonstrated^8,28,31,58^, and activation of ERβ also appears to enhance the effect of androgen deprivation therapy in an experimental system^59^. However, while findings from some preclinical studies are encouraging^16,28^, other findings are conflicting^60,61^ and have reported a lack of effect^60–62^. For ZFHX3, its loss of function is relatively common in advanced prostate cancer, as *ZFHX3* is one of the most frequently mutated genes in prostate cancer; most tumors carrying *ZFHX3* mutations are metastases, high-grade tumors, and/or castration-resistant tumors; and many of the mutations are truncating mutations^19,33,34^. Our finding of ZFHX3 as an indispensable factor for ERβ function in this study suggests that the status of ZFHX3 needs to be considered when restoring or enhancing ERβ activity via its agonists for the treatment of prostate cancer. Without ZFHX3, ERβ would not be able to suppress cell proliferation and tumor growth. Consistent with this, when ZFHX3 expression is higher, higher ERβ expression significantly correlates with both overall survival and DFS, but when ZFHX3 expression is lower, this correlation is absent (Fig. 6g, h). The same consideration applies to the approach of using ERβ as a predictive biomarker for endocrine treatment, which has also been inconclusive at this time^63^.

ZFHX3 coordinates with multiple hormone signaling pathways. For example, ERα not only regulates the expression of ZFHX3 but also interacts with ZFHX3 to regulate gene expression and cell proliferation in breast cancer cells^39^. Additionally, progesterone signaling upregulates the transcription of *ZFHX3* in breast epithelial cells^40^, and ZFHX3 is in turn essential for Pg-PR) to function in mouse mammary gland development^64^. We have demonstrated in this study that ZFHX3 is indispensable for ERβ signaling to suppress cell proliferation and repress *MYC* transcription. It is thus likely that ZFHX3 is also a regulator of androgen/AR signaling in prostate cancer. Currently, we are testing whether this is the case. We are also dissecting the biochemical basis of ZFHX3-ERβ/ERα/PR interactions.

In summary, we examined the relationship between two established transcription factors that are not only essential for normal prostate development but are also tumor suppressors in prostatic tumorigenesis, ERβ and ZFHX3. Using AR-positive prostate cancer cell lines, in which the suppressive activities of ERβ in cell proliferation and MYC expression were validated, we demonstrated that loss of ZFHX3 also increased cell proliferation and MYC expression, and downregulation of MYC was necessary for ZFHX3 to inhibit cell proliferation. Importantly, loss of ZFHX3 prevented ERβ from suppressing cell proliferation and repressing *MYC* transcription, and the necessity for ZFHX3 was due to its interaction with ERβ and their binding to the promoter of *MYC* (Fig. 6f). These findings provide novel insights into the development and progression of prostate cancer.

## Materials and methods

### Cell culture, transfection, and reagents

Human embryonic kidney 293T cells, purchased from ATCC (Manassas, VA, USA), were cultured in Dulbecco’s modified Eagle’s mMedium medium (Gibco, Waltham, MA, USA) supplemented with 10% fetal bovine serum (FBS; Gibco). Human prostate cancer cell lines C4-2B (gift of Dr. Leland Chung, Cedar Sinai Medical Center, VA, USA) and LNCaP (purchased from ATCC) were cultured in RPMI-1640 medium (Gibco) supplemented with 10% FBS. The identities of LNCaP, C4-2B, and clones derived from C4-2B were confirmed by short tandem repeat (STR) analysis. During experiments, cells recovered from a liquid nitrogen freezer were used within 2 months (<20 passages) with no noticeable morphological changes. For all experiments involving DPN or PHTPP treatments, the medium was replaced with phenol red-free medium containing 5% charcoal-stripped FBS 24 h before transfection. The same medium was replaced 24 h after transfection. DPN and PHTPP were purchased from Abcam (Cambridge, MA, USA), soft agar from Lonza (Rockland, ME, USA), and Matrigel from BD Biosciences (Bedford, MA, USA).

For gene silencing by RNAi, cells were transiently transfected with siRNAs using the Lipofectamine RNAiMAX reagent according to the manufacturer’s instructions (Invitrogen, Carlsbad, CA, USA). We used siRNAs from Invitrogen for the knockdown of *ESR2* (RNA-Stealth Select Oligos set, Cat# 1299003). For *MYC* silencing, two siRNAs (Supplementary Table 1) were synthesized by Sangon Biotech (Shanghai, China). The efficiency of RNAi was evaluated by Western blotting (Supplementary Figure S1). Among the three siRNAs against *ESR2*, siESR-2 showed the highest efficiency of knockdown and was used throughout the study. For the two siRNAs against *MYC*, both were effective, and MYC-1 was used in other experiments. The siRNA against ZFHX3 was from a previous study^39^.

Plasmid transfection was performed with Lipofectamine 2000 (Invitrogen).

### Plasmids

Mammalian expression plasmids for pCDNA3-FLAG-ERβ and promoter plasmid for pGL3-MYC and pGL3-MYC-1 were generated using a PCR-based approach with primers listed in Supplementary Table 2. The pGL3-MYC-2 was kindly provided by Dr. Lihong Ye of Nankai University.

### Cell proliferation assay

C4-2B cells were seeded into 24-well tissue culture plates at 5 × 10^4^ cells per well and collected every day or every 3 days. The cells were fixed with 10% trichloroacetic acid for 1 h at 4 °C, washed three times with distilled water, and stained with 100 µl SRB solution (0.4% SRB diluted in 1% acetic acid) for 1 h. Afterwards, plates were washed three times with 1% acetic acid and air dried. The stained cells were dissolved with 10 mM Tris-HCl buffer (pH 8.5), and absorbance was measured. Experiments were performed in triplicate.

### Soft agar colony formation assay

Following previously published procedures^22,65^, C4-2B and LNCaP cells were cultured in RPMI-1640 medium with 10% FBS or phenol red-free RPMI-1640 with 2–5% charcoal-stripped FBS. Two to five thousand cells were suspended in 0.35% agar with or without DPN, and layered on top of 1.5 mL of RPMI-1640 solidified with 0.6% agar in each well of a 6-well plate. After incubation at 37 °C in an CO_2_ incubator for 2 weeks, colonies with a >100 μm were imaged and counted with the ImageJ program. The assay was conducted in triplicate in each experiment, and each experiment was repeated twice.

### Sphere formation assay

The sphere formation assay has been previously described^66^. Briefly, 40 μL of growth factor reduced Matrigel was added to each well of 8-well glass chamber slides and spread evenly. After Matrigel was solidified for 15 min at 37 °C, 400 μL of cell suspension (2000 cells) in RPMI-1640 medium containing 10% FBS and 2% Matrigel was seeded into each well, and the medium was replaced every 3 days. Images of spheres with defined sizes were subjected to the ImageJ computer program. Spheres with a diameter larger than 75 μm were counted.

### Immunoprecipitation and Western blotting

Cultured cells with indicated treatments were washed twice with ice-cold phosphate-buffered saline (PBS) and then lysed in modified radioimmune precipitation assay buffer (150 mM NaCl, 50 mM Tris-HCl, pH 7.5, 1% NP-40, and protease inhibitor mixture). Cell lysates were centrifuged to collect supernatants, which were first incubated overnight with different antibodies and then with protein A/G-agarose (Invitrogen) with rotation at 4 °C for another 2 h. After washing three times with modified radioimmune precipitation assay buffer, immunoprecipitates were released by boiling for 10 min in 50 μL loading buffer, resolved in 4–10% sodium dodecyl sulfate-polyacrylamide gel electrophoresis, and then blotted with different antibodies (details of the antibodies are listed in Supplementary Table 3). Horseradish peroxidase- conjugated secondary antibodies and goat anti-rabbit immunoglobulin G (IgG) or goat anti-mouse IgG were diluted at 1:5000. Signals were detected by Western Bright ECL (Advansta, Menlo Park, CA, USA), and blots were photographed with the luminescent image analyzer (Jun Yi Dong Fang, Beijing, China).

### Genome engineering for ZFHX3 truncation

ZFHX3-specific single guide RNA (sgRNA) oligos were designed following the published protocol^67^ and cloned into the LentiCRISPR, which was kindly provided by Dr. Yushan Zhu. The following pairs of sgRNA were used: 5′-CACCGGGCAGATCTTCACCATCCGC-3′ (forward) and 5′-AAACGCGGATGGTGAAGATCTGCCC-3′ (reverse). Lentiviral particles were produced in 293T cells by co-transfecting pLKO.1 with pMD2.G and psPAX2 plasmids using the FuGENE 6 transfection reagent (Promega, Madison, WI, USA) according to the manufacturer’s protocol. Six hours after transfection, the medium was replaced, and cells were incubated for an additional 2 days before viral supernatant was collected. Human C4-2B cells were seeded in 6-well culture plates and grown to about 70% confluency. Culture medium was then replaced with 2 mL of fresh medium containing 8 µg/mL of polybrene and 1 mL of lentiviral supernatant. Six to 12 h after viral infection, the lentivirus-containing medium was replaced with fresh medium containing puromycin (Sigma, St Louis, MO, USA) at 3 µg/mL, and the incubation continued for 3–5 days to select cells stably expressing sgRNAs. Cells were then seeded into 96-well plates at 0.5 cells/well, and single-cell clones were collected and identified by DNA sequencing and Western blotting analysis.

### Chromatin IP assay

C4-2B cells were grown for 3 days in phenol red-free RPMI-1640 medium supplemented with 2% charcoal-stripped FBS and treated with either vehicle or 100 nM DPN for 45 min. ChIP assay was performed according to the manufacturer’s instruction using the SimpleChIP Enzymatic Chromatin IP Kit (Magnetic Beads) from Cell Signaling Technology (Danvers, MA, USA). Briefly, cells were cross-linked with 1% formaldehyde for 10 min at room temperature. Glycine quenched samples were washed with ice-cold PBS. Cells were then lysed, and nuclei were separated. Micrococcal nuclease was added to digest DNA for 20 min at 37 °C, and digestion reactions were stopped by adding 0.5 M EDTA. The nuclear pellet was collected and incubated in ChIP buffer with protease inhibitors for 10 min on ice. Sheared cross-linked chromatin preparation was collected after sonication. Chromatin extracts were immunoprecipitated by ZFHX3 or ERβ antibody using normal rabbit IgG or mouse IgG as a negative control. Quantitative real-time PCR was performed using the Realplex real-time PCR detection system (Eppendorf, Hamburg, Germany). Sequences of primers are described in Supplementary Table 4.

### Reverse transcription and real-time PCR

Total RNA was isolated using the TRIzol reagent (Invitrogen) according to the manufacturer’s protocol and was used for complementary DNA synthesis with the Moloney murine leukemia virus reverse transcriptase system (Promega). Real-time PCR was performed using the Mastercycler ep Realplex system (Eppendorf) using the SYBR premix Ex Taq (TaKaRa Bio Inc., Tianjin, China). Primer sequences were as follows: 5′-GGTGGTCTCCTCTGACTTCAACA-3′ (*GAPDH* forward), 5′-GTTGCTGTAGCCAAATTCGTTGT-3′ (*GAPDH* reverse), 5′-GTCAAGAGGCGAACACACAAC-3′ (*MYC* forward), and 5′-TTGGACGGACAGGATGTATGC-3′ (*MYC* reverse).

### Luciferase reporter gene assays

C4-2B cells were transiently transfected with pGL3, pGL3-MYC, or pGL3-MYC-1/2 plasmid and the pRT-TK Renilla luciferase plasmid (Promega). Luciferase activities were determined 48 h after transfection and DPN treatments using the Dual-Luciferase Reporter Gene Assay Kit (Promega). Luciferase activity was normalized to Renilla luciferase activity in each reaction. Experiments were performed in triplicate.

### Correlation between *ZFHX3*/*ESR2* expression and patient survival and other statistical analyses

Two previously published cohorts of prostate cancer patients were used for survival analysis. One included 281 men who either died of prostate cancer or survived for more than 10 years without metastases^51^, and the other included 140 prostate cancers that had both mRNA expression data and disease-free survival status^52^. Survival curves were prepared by using the Kaplan–Meier analysis, and the statistical parameters were calculated by a log-rank test.

All experiments were repeated at least twice, unless stated otherwise. All experimental readings were expressed as mean ± standard errors. Means were compared with one-way analysis of variance or two-way analysis of variance when applicable. Multiple comparisons were performed by Tukey’s multiple comparisons test and *P* values < 0.05 were considered as statistically significant. All statistical analyses were conducted using the SPSS 21 package (IBM Analytics, Armonk, NY, USA).

## Supplementary information

Supplementary legends

Supplementary Figure S1

Supplementary Table 1

Supplementary Table 2

Supplementary Table 3

Supplementary Table 4

C4-2B STR analysis

Wt STR analysis

KO3 STR analysis

KO8 STR analysis

LNCaP STR analysis

original western blot
