# Supplementary material for: ZFHX3 is indispensable for ERβ to inhibit cell proliferation via MYC downregulation in prostate cancer cells
Source: Oncogenesis. 2019 Apr 12;8(4):28. doi: 10.1038/s41389-019-0138-y (PMC6461672; doi:10.1038/s41389-019-0138-y)
Supplement: Supplementary file 1 — Supplementary legends [file 41389_2019_138_MOESM1_ESM.docx]

Supplementary figure

**Supplementary Figure S1. Loss of ZFHX3 increased MYC and FOXO3A expression and efficiencies of ERβ and MYC knockdown in prostate cancer cells.** (a) Knockout of *ZFHX3* upregulated MYC and FOXO3A expression in C4-2B cells, as detected by Western blotting. Wt and KO8 are vector control and ZFHX3-null clones of C4-2B respectively. (b, c) C4-2B and LNCaP cells were transfected with siRNAs against *ESR2* (b) and *MYC* (c), and expression of ERβ and MYC was detected by Western blotting.
