# Supplementary figures and images for: ZFHX3 is indispensable for ERβ to inhibit cell proliferation via MYC downregulation in prostate cancer cells
Source: Oncogenesis. 2019 Apr 12;8(4):28. doi: 10.1038/s41389-019-0138-y (PMC6461672; doi:10.1038/s41389-019-0138-y)

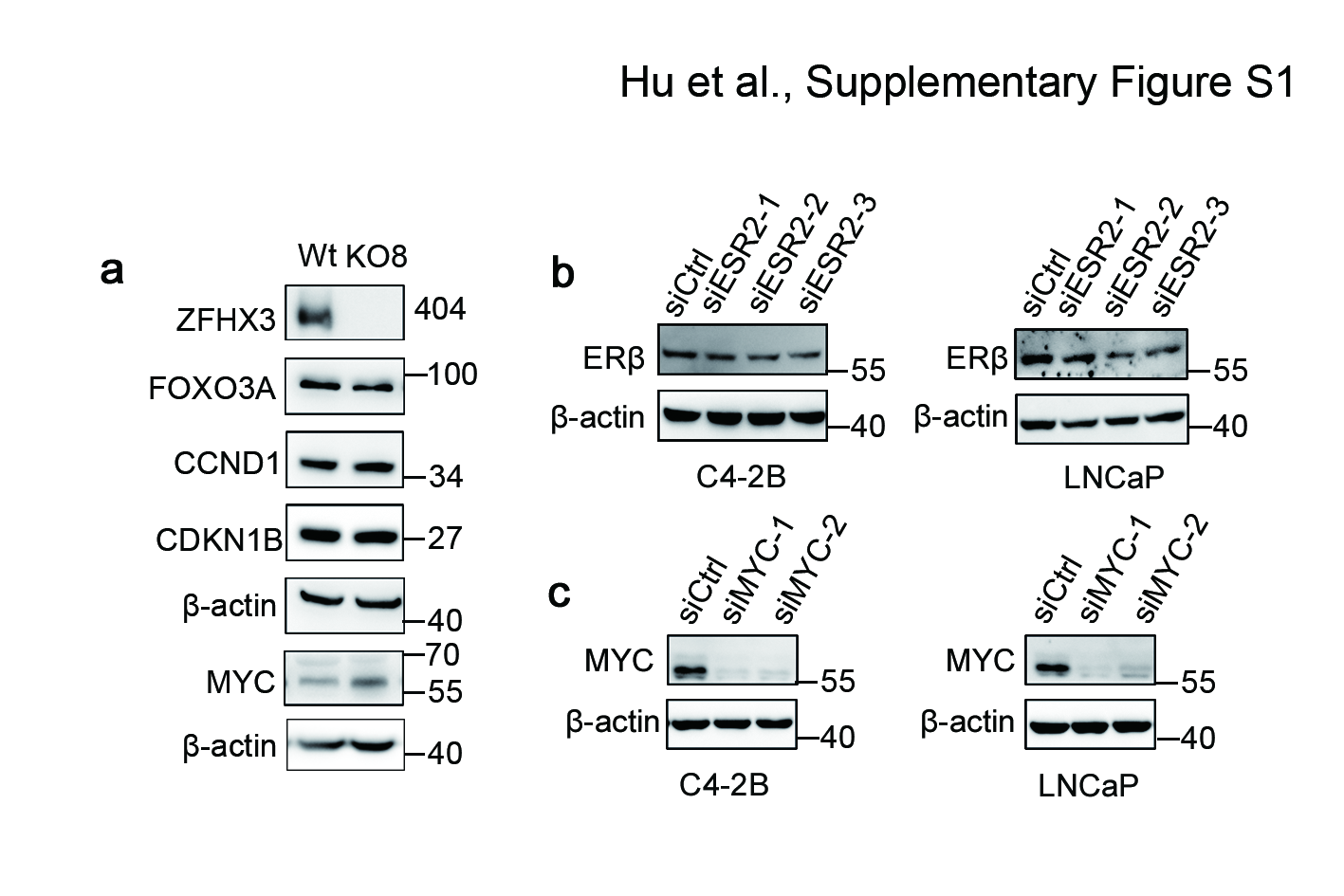

Supplement: Supplementary file 2 — Supplementary Figure S1 [file 41389_2019_138_MOESM2_ESM.tif]

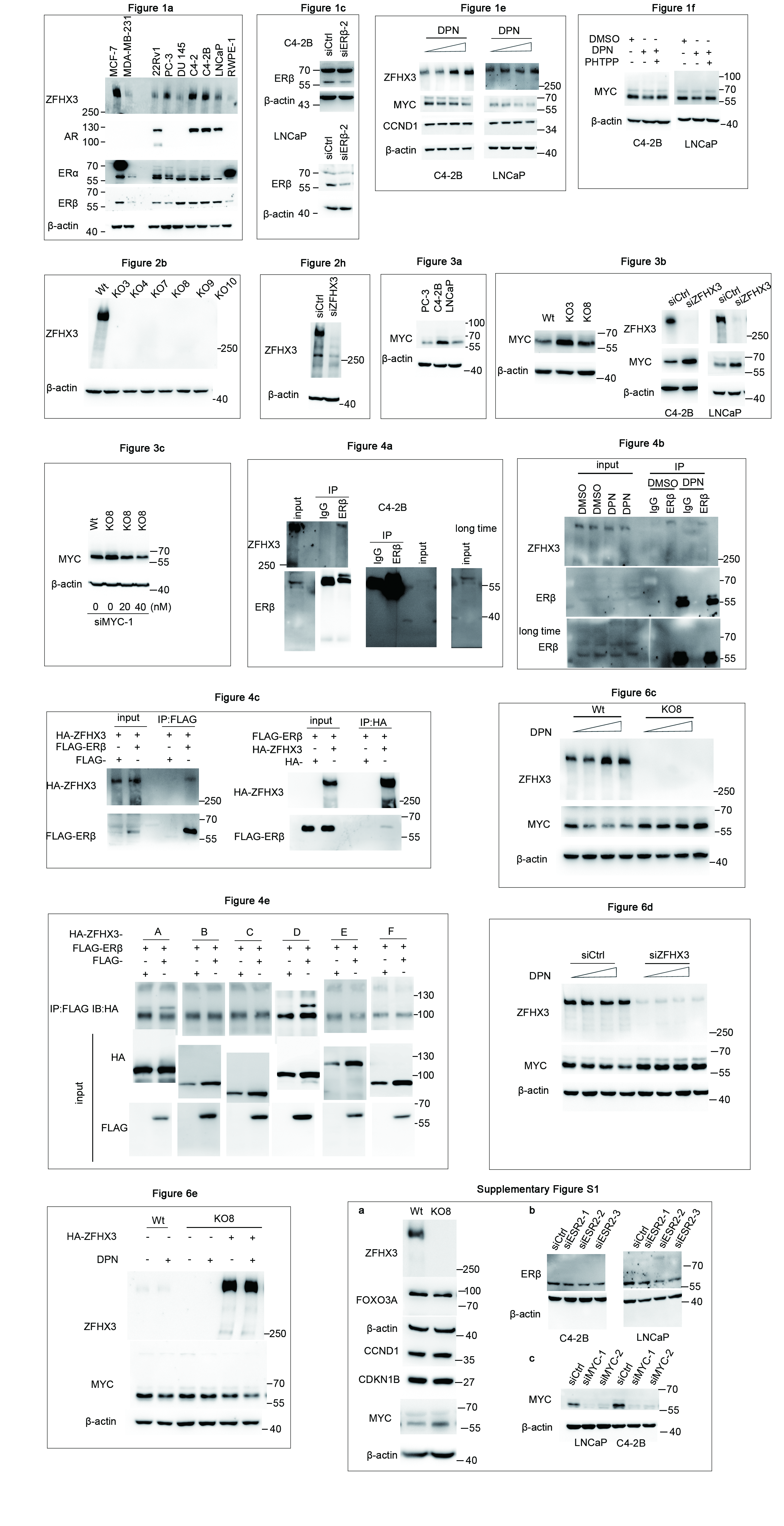

Supplement: Supplementary file 12 — original western blot [file 41389_2019_138_MOESM12_ESM.tif]
