# Supplementary material for: ZFHX3 is indispensable for ERβ to inhibit cell proliferation via MYC downregulation in prostate cancer cells
Source: Oncogenesis. 2019 Apr 12;8(4):28. doi: 10.1038/s41389-019-0138-y (PMC6461672; doi:10.1038/s41389-019-0138-y)
Supplement: Supplementary file 3 — Supplementary Table 1 [file 41389_2019_138_MOESM3_ESM.docx]

| **Names** | **Sequence** |
| --- | --- |
| ESR2-1 | CACTTCTGCGCTGTCTGCAGCGATT |
| ESR2-2 | CCCTGCTGTGATGAATTACAGCATT |
| ESR2-3 | CCTTTAGTGGTCCATCGCCAGTTAT |
| MYC-1 | GGAACUAUGACCUCGACUA |
| MYC-2 | GAACACACAACGUCUUGGA |

**Supplementary Table 1: Target sequences of siRNAs**
