# Supplementary material for: ZFHX3 is indispensable for ERβ to inhibit cell proliferation via MYC downregulation in prostate cancer cells
Source: Oncogenesis. 2019 Apr 12;8(4):28. doi: 10.1038/s41389-019-0138-y (PMC6461672; doi:10.1038/s41389-019-0138-y)
Supplement: Supplementary file 4 — Supplementary Table 2 [file 41389_2019_138_MOESM4_ESM.docx]

**Supplementary Table 2: Primer sequences used in gene clone**

| **Names** | **Forward** | **Reverse** |
| --- | --- | --- |
| ERβ | CGGGATCCGGATGGATATAAAAAACTCACC | CGTCTAGATCACTGAGACTGTGGGTTCTGGG |
| pGL3-MYC | CCGAGCTCAGGGAAAGACGCTTTGCA | GGAAGATCTGCGGTCACCATCTCCAG |
| pGL3-MYC -1 | CCGAGCTCGCAACTAGCTAAGTCGAAGC | GGAAGATCTGCCGTTCAGAGCGTGGGAT |
