# Supplementary material for: ZFHX3 is indispensable for ERβ to inhibit cell proliferation via MYC downregulation in prostate cancer cells
Source: Oncogenesis. 2019 Apr 12;8(4):28. doi: 10.1038/s41389-019-0138-y (PMC6461672; doi:10.1038/s41389-019-0138-y)
Supplement: Supplementary file 5 — Supplementary Table 3 [file 41389_2019_138_MOESM5_ESM.docx]

| **Antibodies** | **Company** | **Catalog No.** | **Dilution** |
| --- | --- | --- | --- |
| ZFHX3 | Self-manufacture | NA | 1:800 |
| β-actin | Sigma Aldrich | A1978 | 1:5000 |
| ERβ (CHIP and IP) | Abcam | Ab288 | 1:1000 |
| ERβ (Immunoblot) | GeneTex | GTX112927 | 1:1000 |
| MYC | Cell Signaling | 9402S | 1:1000 |
| AR | Cell Signaling | 5153S | 1:2000 |
| ERα | Santa Cruz | 8005 | 1:2000 |
| CCND1 | Abcam | 134175 | 1:10,000 |
| FLAG | Sigma | F1804 | 1:3000 |
| HA | Cell Signaling | 3724S | 1:3000 |
| FOXO3A | Cell Signaling | 2497S | 1:2000 |
| CDK1B | BD | 610241 | 1:1000 |

**Supplementary Table 3: Antibodies**
