# Supplementary material for: ZFHX3 is indispensable for ERβ to inhibit cell proliferation via MYC downregulation in prostate cancer cells
Source: Oncogenesis. 2019 Apr 12;8(4):28. doi: 10.1038/s41389-019-0138-y (PMC6461672; doi:10.1038/s41389-019-0138-y)
Supplement: Supplementary file 6 — Supplementary Table 4 [file 41389_2019_138_MOESM6_ESM.docx]

**Supplementary Table 4 : Primer sequences used in CHIP**

| **Primer** | **Forward** | **Reverse** |
| --- | --- | --- |
| Region A | CCAGATAGCTGTGCATACAT | GGAGAGTGGAGGAAAGAAGGGT |
| Region B | CTGGGACTCTTGATCAAAGC | CCTGTGAGTATAAATCATCGCAGG |
| Region C | TGCGATGATTTATACTCACAG | AGGTGGGGAGGAGACTCAGCCGG |
| β-actin | CGGAGGGCGCCCCAACTCAG | GCGCGCGCGGCCCCAGAACA |
