# Supplementary material for: ZFHX3 is indispensable for ERβ to inhibit cell proliferation via MYC downregulation in prostate cancer cells
Source: Oncogenesis. 2019 Apr 12;8(4):28. doi: 10.1038/s41389-019-0138-y (PMC6461672; doi:10.1038/s41389-019-0138-y)
Supplement: Supplementary file 8 — Wt STR analysis [file 41389_2019_138_MOESM8_ESM.pdf]

GENEWIZ®

Solid science. Superior service.

Cell Line Authentication Report

GENEWIZ, Inc.

C3 Building, 218 Xinghu Road

Suzhou Industrial Park, 215123

Suzhou, China

Tel: 400-8100-669

www.genewiz.com

Cell Line Authentication Report

Customer: Qingxia hu

Institution: Southern University of Science and Technology

Quotation Number: 80-269564405

Completion Date：3/6/2019

1. **Sample ID: Wt**
2. **Original Material: Cell pellets**
3. **Methods:**

1).Genomic DNA was extracted from the cell pellets provided by the customer.

2).Samples, together with positive and negative control were amplified using GenePrint 10 System (Promega).

3). Amplified products were processed using the ABI3730xl Genetic Analyzer.

4). Data were analyzed using GeneMapper4.0 software and then compared with the ATCC, DSMZ, JCRB and RIKEN etc. databases for reference matching.

4. **Results:**

1) 10 Loci STR Profile：

<table>
<tr><td>Genetic Site</td><td colspan="3">Customer sample</td></tr>
<tr><td>(Locus)</td><td colspan="3">Wt</td></tr>
<tr><td>Amelogenin</td><td>X</td><td colspan="2">X</td></tr>
<tr><td>CSF1PO</td><td>9</td><td>10</td><td>11</td></tr>
<tr><td>D13S317</td><td>10</td><td colspan="2">11</td></tr>
<tr><td>D16S539</td><td>11</td><td colspan="2">11</td></tr>
<tr><td>D5S818</td><td>11</td><td colspan="2">12</td></tr>
<tr><td>D7S820</td><td>9.1</td><td colspan="2">10.3</td></tr>
<tr><td>THO1</td><td>9</td><td colspan="2">9</td></tr>
<tr><td>TPOX</td><td>8</td><td colspan="2">9</td></tr>
<tr><td>vWA</td><td>16</td><td colspan="2">18</td></tr>
<tr><td>D21S11</td><td>29</td><td colspan="2">32.2</td></tr>
</table>

GENEWIZ

<<< Search for reference matching with the Cell Bank databases and add the match results.

Result of STR matching analysis by your data.

- DSMZ Profile Database -

A graphical presentation is shown at the bottom of this page.

EV	Cell No.	Cell name	Locus names									Figures
			D5S818	D13S317	D7S820	D16S539	VWA	TH01	AM	TPOX	CSF1PO	
	Query (Your Cell)		11,12	10,11	9.1,10.3	11,11	16,18	9,9	X,X	8,9	9,10,11	
0.86(32/37)	256	LNCAP	11,12	10,12	9.1,10.3	11,11	16,18	9,9	X,Y	8,9	10,11	-
0.86(32/37)	CRL-1740	LNCaP clone FGC	11,12	10,12	9.1,10.3	11,11	16,18	9,9	X,Y	8,9	10,11	-
0.74(28/38)	RCB2144	LNCap.FGC	11,12	10,12	9,9	11,11	16,17,18	9,9	X,Y	8,9	10,11	-
0.65(24/37)	104	M-07e	11,11	10,11	11,11	11,11	16,18	6,8	X,X	8,8	9,10	-
0.65(24/37)	CRL-3035	CHLA-03-AA	11,11	10,11	9,10	11,12	16,18	6,9	X,X	8,9	10,10	-
0.65(24/37)	RCB2141	PK-45P	10,12	9,11	11,11	11,12	16,18	9,9	X,X	8,11	9,10	-
0.65(24/37)	RCB2266	HE50	11,12	10,12	8,9	11,11	17,17	9,9	X,Y	8,9	10,11	-
0.63(26/41)	140	SUP-T1	11,12,10	10,11,12	11,11	9,10,11	16,18,19	9.3,9.3	X,X	8,9	10,11	-
0.62(26/42)	759	LS-174T	11,15,14	10,11,9	10.3,11	11,12,13	15,18,16	6,7	X,X	8,9	10,14,11	-

>>>

GENEWIZ

GENEWIZ, Inc.Suzhou
C3 Building, 218 Xinghu Road
Suzhou Industrial Park, 215123
Suzhou, China
+86 400-8100-669

2) Electrophoretogram

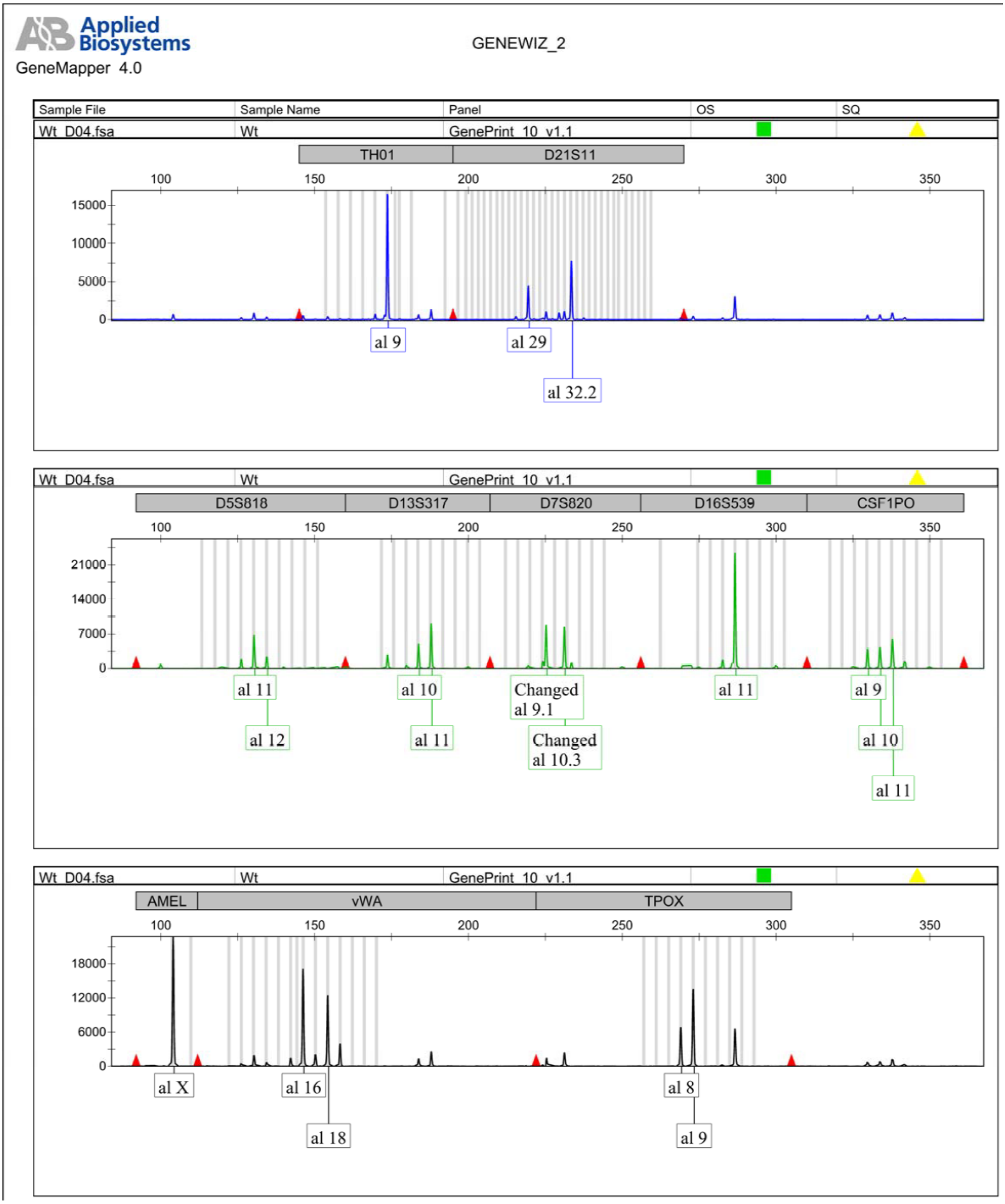

Note：Raw data in appendix
