# Supplementary material for: ZFHX3 is indispensable for ERβ to inhibit cell proliferation via MYC downregulation in prostate cancer cells
Source: Oncogenesis. 2019 Apr 12;8(4):28. doi: 10.1038/s41389-019-0138-y (PMC6461672; doi:10.1038/s41389-019-0138-y)
Supplement: Supplementary file 11 — LNCaP STR analysis [file 41389_2019_138_MOESM11_ESM.pdf]

Solid science. Superior service.

Cell Line Authentication Report

GENEWIZ, Inc.

C3 Building, 218 Xinghu Road

Suzhou Industrial Park, 215123

Suzhou, China

Tel: 400-8100-669

www.genewiz.com

GENEWIZ, Inc.Suzhou
C3 Building, 218 Xinghu Road
Suzhou Industrial Park, 215123
Suzhou, China
+86 400-8100-669

Cell Line Authentication Report

Customer: Qingxia hu

Institution: Southern University of Science and Technology

Quotation Number: 80-269564405

Completion Date： 3/6/2019

1. **Sample ID: LNCaP**
2. **Original Material: Cell pellets**
3. **Methods:**

1).Genomic DNA was extracted from the cell pellets provided by the customer.

2).Samples, together with positive and negative control were amplified using GenePrint 10 System (Promega).

3). Amplified products were processed using the ABI3730xl Genetic Analyzer.

4). Data were analyzed using GeneMapper4.0 software and then compared with the ATCC, DSMZ, JCRB and RIKEN etc. databases for reference matching.

4. **Results:**

1) 10 Loci STR Profile：

Genetic Site	Customer sample	
(Locus)	LNCaP	
Amelogenin	X	Y
CSF1PO	10	11
D13S317	10	12
D16S539	11	11
D5S818	11	12
D7S820	9.1	10.3
THO1	9	9
TPOX	8	9
vWA	16	18
D21S11	29	32.2

GENEWIZ

<<< Search for reference matching with the Cell Bank databases and add the match results.

Result of STR matching analysis by your data.

- DSMZ Profile Database -

A graphical presentation is shown at the bottom of this page.

EV	Cell No.	Cell name	Locus names									Figures
			D5S818	D13S317	D7S820	D16S539	VWA	TH01	AM	TPOX	CSF1PO	
	Query (Your Cell)		*11,12*	*10,12*	*9.1,10.3*	*11,11*	*16,18*	*9,9*	*X,Y*	*8,9*	*10,11*	
1.00(36/36)	256	LNCAP	11,12	10,12	9.1,10.3	11,11	16,18	9,9	X,Y	8,9	10,11	-
1.00(36/36)	CRL-1740	LNCaP clone FGC	11,12	10,12	9.1,10.3	11,11	16,18	9,9	X,Y	8,9	10,11	-
0.86(32/37)	RCB2144	LNCap.FGC	11,12	10,12	9,9	11,11	16,17,18	9,9	X,Y	8,9	10,11	-
0.78(28/36)	RCB2266	HE50	11,12	10,12	8,9	11,11	17,17	9,9	X,Y	8,9	10,11	-
0.67(24/36)	CRL-2220	CA-HPV-10	11,12	11,12	10,11	11,11	16,17	6,8	X,Y	8,9	10,11	-
0.67(24/36)	JCRB0041	HLCL-1	11,13	10,12	8,12	11,11	16,17	9,9	X,Y	9,11	11,12	-
0.61(22/36)	237	IGR-37	11,12	12,12	10,11	11,11	17,21	9,9	X,Y	8,11	11,12	-
0.61(22/36)	435	HSB-2	11,12	10,12	11,12	9,12	18,19	9,10	X,Y	8,8	10,11	-
0.61(22/36)	676	NGP	11,12	8,12	11,11	8,11	16,21	9,9	X,Y	6,9	11,12	-
0.61(22/36)	714	KASUMI-3	12,12	11,12	10,11	11,11	16,18	6,9	X,Y	8,8	11,11	-
0.61(22/36)	CRL-1110	Sal Mat	11,12	9,12	8,11	11,12	16,18	7,9	X,Y	7,8	7,11	-
0.61(22/36)	CRL-1834	GK-5	10,12	12,12	10,10	11,11	16,19	9,9.3	X,Y	8,8	10,11	-
0.61(22/36)	CRL-7062	Hs 93.T	10,11	11,12	11,13	11,11	14,16	7,9	X,Y	8,9	10,12	-

>>>

2) Electrophoretogram

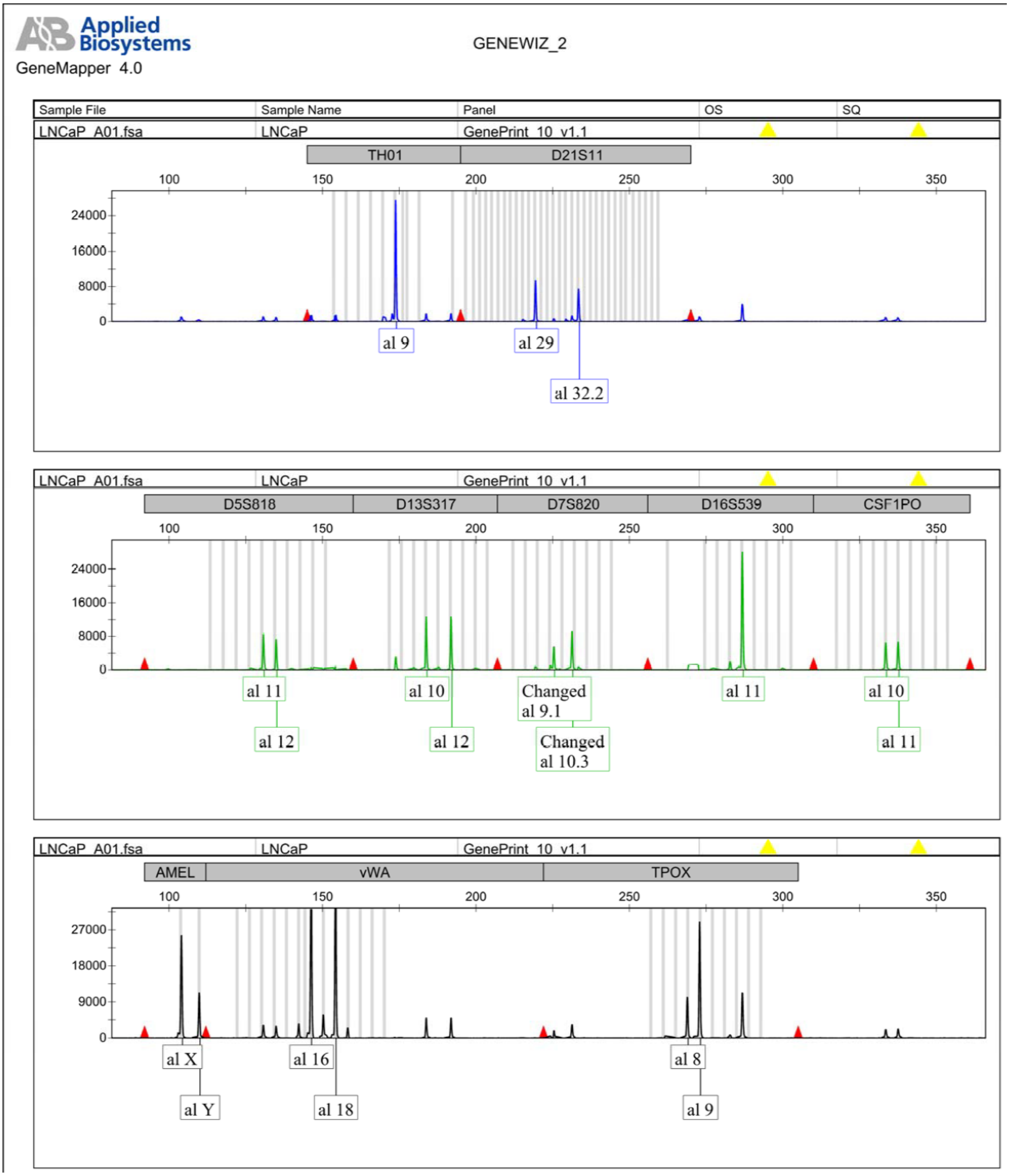

Note：Raw data in appendix
